# The Core Needle and Surgical Biopsy Concordance to Detect Estrogen, Progesterone, and Her-2 Receptors in Breast Cancer: A Comparative Study

**Published:** 2017-07-01

**Authors:** Fereshteh Ensani, Ramesh Omranipour, Isa Jahanzad, Azadeh Jafari, Shima Nafarzadeh, Pouyan Aminishakib

**Affiliations:** 1 *Dept. of Pathology, Cancer Institute Hospital, Tehran University of Medical Sciences, Tehran, Iran*; 2 *Surgical Oncology Center Institute Hospital, Tehran University of Medical Science, Tehran, Iran*; 3 *Dept. of Immunohistochemistry, Cancer Institute, Tehran University of Medical Science, Tehran, Iran*; 4 *Dept. of Oral and Maxillofacial Pathology, Babol University of Medical Sciences, Tehran, Iran*; 5 *Dept. of Oral and Maxillofacial Pathology, Tehran University of Medical Sciences, Tehran, Iran*

**Keywords:** Core Needle Biopsy, Breast Cancer, Estrogen, Progesterone, Her-2

## Abstract

**Background &Objectives:**

Evaluation of estrogen receptor (ER), progesterone receptor (PR), and (human epidermal growth factor receptor-2) Her-2 on core needle biopsies (CNBs) is increasingly in use to diagnosis early breast cancer, but its concordance with surgical excision (SE) is not well documented.

**Methods:**

The study included 100 formalin fixed, paraffin-embedded specimens of invasive breast carcinoma archived in Pathology Department of Cancer Institute, Tehran, Iran, from 2011 to 2014. Immunohistochemistry was applied to detect ER, PR, and Her-2.

**Results:**

The current study findings indicated a significant correlation of 90% between CNB and SE specimens for *ER* expression. The correlation between CNB and SE specimens was estimated as 81% and 97.3% for PR and Her-2, respectively.

**Discussion:**

CNB can be performed confidently to determine ER and Her-2. For PR, results obtained from CNB should be considered**.**

## Introduction

Breast cancer is one of the most frequently diagnosed neoplasms among females (1), and also one of the main causes of the mortality and morbidity worldwide (1,2,3). In Iran, it is estimated that breast cancer affects 20 new cases in every 1000 females annually, and studies show that Iranian females develop breast cancer in a younger ages, 35 to 44 years old, compared with those of the Western countries (4).

Nowadays, increasing screening programs lead to the discovery of clinically occult lesions (3), and death rates decrease due to earlier detection and more effective treatments (4). Since the prediction of tumor behavior is a crucial component for long term follow-up treatment plans, finding clinical or pathologic prognostic parameters is of great value (5).

CNB has an undeniable role to diagnose both palpable and impalpable lesions (1,2,6), and provides substantial information to determine the subsequent therapeutic strategies due to the immunohistochemical evaluations of CNB, particularly in the setting of neo adjuvant therapy (6,7). It is an inevitable practical benefit to avoid further unnecessary surgical biopsies (8). Based on the possibility of heterogeneous distribution of hormonal receptors within tissue, the need for a reliable procedure to accurately depict the tumor hormonal profile arises, and still there is a great debate about the more effective procedures (1,6).

In addition to histopathologic findings, prognostic factors status including estrogen receptor (ER), progesterone receptor (PR) have essential roles in ascertaining an effective treatment plan (1,2,9,10). *ER* and *PR* expression profiles are introduced as important parts of breast cancer pathologic evaluation as their statuses describe the ensuing hormonal treatment efficacy (11).

On the other hand, although the overexpression of Her-2 (human epidermal growth factor receptor-2) is associated with patients’ worse prognosis (1), recently developed drugs for targeted therapy and combination of trastuzumab with chemotherapy are introduced as effective treatments for patients with Her-2 positive breast cancers (12,13).

Previous studies showed variable consistency rates of hormonal receptor status between CNBs and surgically excised specimens (14). Therefore, the current study aimed at comparing ER, PR, and Her-2 statuses between CNBs and surgically excised specimens.

## Material and Methods

**Samples**

The current cross sectional, retrospective study was conducted in the second half of 2015 on a total of 100 formalin fixed, paraffin-embedded specimens definitely diagnosed as invasive breast carcinoma archived in Pathology Department of Cancer Institute, Tehran, Iran,from2011 to 2014.Thestudy was approved by the Ethics Committee of Tehran University of Medical Sciences, and the routine paraffin block-included study rules, such as preservation of adequate specimen for further evaluations, were considered. Both lobular and ductal carcinoma with different grades (1,2,3) were included in the study. Patients' data including age and number of CNB specimens were collected from the archive. All of the included patients had a CNB followed by a wide surgical excision (lumpectomy or mastectomy). Neo adjuvant therapy, metastasis and recurrence were considered as exclusion criteria. In order to confirm the diagnosis, hematoxylin and eosin stained slides of all samples were prepared and evaluated. Then, immunohistochemical staining method was employed to detect *ER*, *PR*, and *Her-2* expression. 

**Immunohistochemical staining**

Briefly, 5-µm thick sections, prepared from samples, were incubated at37ºCand 54ºC for 24 hours and 30 minutes, respectively. Slides were deparaffinized and rehydrated using xylene and ethanol 100%, 96% and 70%, and incubated with 0.5% H_2_O_2_ in methanol for 20 minutes to block the endogenous peroxidase activity. Then, slides were washed with distilled water and heated for 20 minutes at 120ºCwith citrate buffer in an autoclave to retrieve the antigens. Slides were, then, put in phosphate-buffered saline (PBS), H_2_O_2_, distilled water, and again PBS, respectively. Next step was incubation with ready-to-use primary antibodies (1D5 for ER, PR PR88 for PR, and anti-Her2 EP1045Y from Bio Genex, Fremont, CA). Then, Novo link Polymer RE 7150-k kit was used for horse anti-mouse IgG secondary antibodies and 3,3'-diaminobenzidine(DAB) as chromogen. Washing with Tris-buffered saline (TBS) was done after each step. Finally, counter staining with the Mayer hematoxylin, dehydration and mounting with DPX mountant were performed. The current study used normal breast tissue as positive controls, and negative controls were obtained by replacing the primary antibody with nonimmune IgG. 

**Immunohistochemical staining evaluation**

Microscopic evaluation was performed by two independent general pathologists using light microscope (Olympus BX41, Tokyo, Japan). Percentage of stained cells (nucleus staining) in five high power fields were figured, and quick score system was used to evaluate *ER* and *PR* expression as follow: no staining (0), 1% to 25% stained cells (1), 26% to 50% (2), 51% to 75% (3), > 75% (4) (9). For staining intensity, the following categories were considered: no staining (0), weak staining (+), moderate staining (++), and strong staining (+++). For Her-2 expression, samples were divided into the following categories: 10% cells with no staining or weak and incomplete cell membrane staining (-), 10% cells with completely weak to moderate cell membrane staining (equivocal), and>10% cells with circumferential and complete membrane staining (+).

## Results

**Figure 1:**
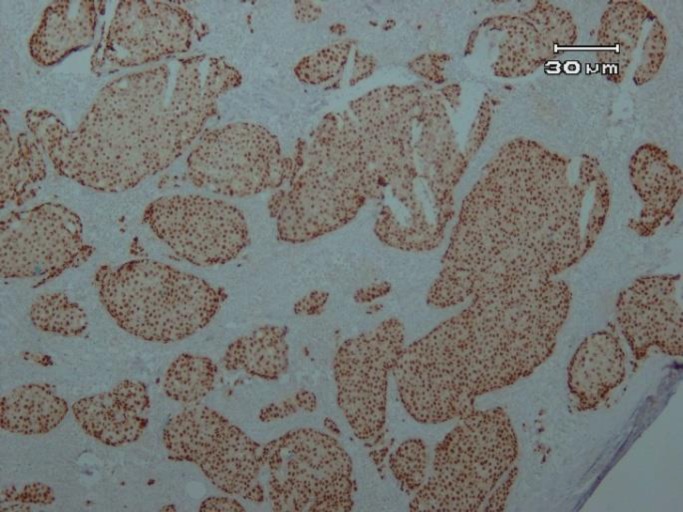
Strong ER staining in more than 75% of tumoral cells (×100)

**Figure 2:**
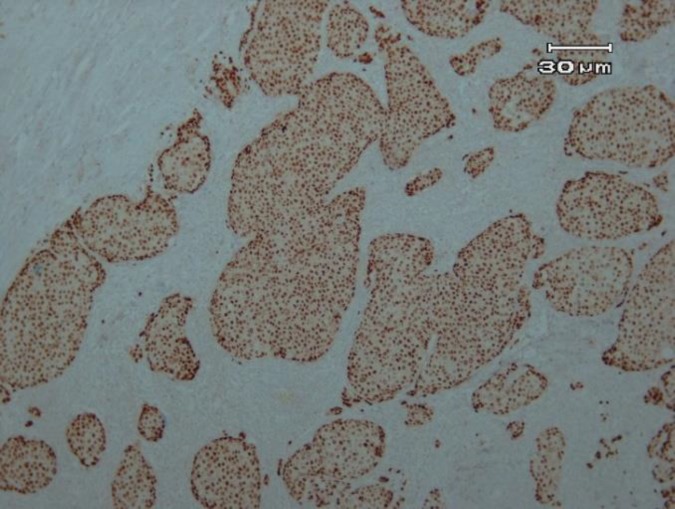
Strong PR staining in more than 75%of tumoral cells (×100)

**Figure 3:**
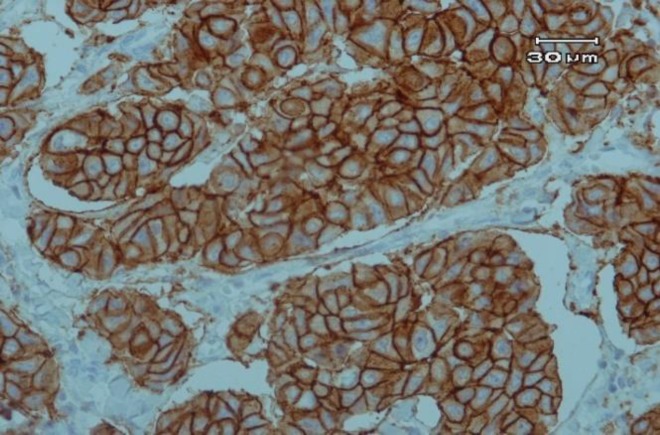
Circumferential and intense, complete staining of Her-2 in more than 10% of tumoral cells (×100).

A total of 100 patients with the mean age of 50.44 years, ranged from 28 to 75 years, were included in the current study. The immunohistochemical expression of *ER*, *PR*, and *Her-2* are shown in figures 1, 2 and 3.

***ER*** **expression in core needle biopsy and surgically excised specimens**

To compare the CNB with SE specimens regarding *ER* expression, 89%of positive CNBs were confirmed by SE specimens and also 92%of negative CNBs were followed by the same findings of SE specimens (Table 1).

**Table 1:** Status of ER in CNB and SE Specimens

			SE		Total
			Negative	Positive	
CNB	Negative	Number	13	1	14
		CNB (%)	92.9	7.1	100.0
		SE (%)	59.1	1.3	14.3
		Total (%)	13.3	1.0	14.3
	Positive	Number	9	75	84
		CNB (%)	10.7	89.3	100.0
		SE (%)	40.9	98.7	85.7
		Total (%)	9.2	76.5	85.7
Total		Number	22	76	98
		CNB (%)	22.4	77.6	100.0

Estrogen receptor; CNB, core needle biopsy; SE, surgical excision

***PR*** **expression in core needle biopsy and surgically excised specimens**

The *PR* expressions of 80.7%in CNB-positive cases were endorsed by SE specimens and additionally, 80%of negative cases were followed by confirmatory SE staining (Table 2)

A significant correlation of 90% was observed between CNB and SE specimens regarding *ER* expression.

The correlation between CNB and SE specimens was estimated as 81% regarding *PR* expression.

***Her-2*** **expression in core needle biopsy and surgically excised specimens**

*Her-2* expression in both positive (100%) and negative (96.7%) cases of CNBs were confirmed by SE specimens (Table 3); a significant correlation of 97.3% was observed between the two methods.

**Table 2:** Status of PR in CNB and SE specimens

	SE	Total			
	Negative	Positive			
CNB	Negative	Number	8	2	10
		CNB (%)	80.0	20.0	100.0
		SE (%)	32.0	2.7	10.2
		Total (%)	8.2	2.0	10.2
	Positive	Number	17	71	88
		CNB (%)	19.3	80.7	100.0
		SE (%)	68.0	97.3	89.8
		Total (%)	17.3	72.4	89.8
Total	Number	25	73	98
	CNB (%)	25.5	74.5	100.0
	SE (%)	100.0	100.0	100.0
	Total (%)	25.5	74.5	100.0

PR, progesterone receptor; CNB, core needle biopsy; SE, surgical excision

## Discussion

CNB is widely performed as a part of the triple assessment in preoperative evaluation of suspected patients of breast cancer. It is more reliable than fine needle aspiration biopsy for histopathologic and IHC evaluations, but there is a great concern that CNB may not be a well representative of SE specimens (15,16).

Hormone receptors including ER, PR, and Her-2 render an essential role as biomarkers in breast carcinoma and stand as keystone for each patient best treatment plans (1,2,3,6).

During recent years, several studies elucidated the concordance of CNB and SE specimens in the evaluation of patients' hormonal profile with a wide spectrum of various findings (14). Some researchers believe in the efficacy of old surgical procedures because of the small specimen size (3), sampling error, and crash artefacts that happen in CNB (13);while, others believe that CNB is the only useful method for candidates of adjuvant therapy (1,2).

**Table 3:** Status of Her-2 in CNB and SE specimens

	SE	Total			
	Negative	Positive			
CNB	Negative	Number	59	2	61
		CNB (%)	96.7	3.3	100.0
		SE (%)	100.0	13.3	82.4
		Total (%)	79.7	2.7	82.4
	Positive	Number	0	13	13
		CNB (%)	0	100.0	100.0
		SE (%)	0	86.7	17.6
		Total (%)	0	17.6	17.6
Total	Number	59	15	14
	CNB (%)	79.7	20.3	100.0
	SE (%)	100.0	100.0	100.0
	Total (%)	79.7	20.3	100.0

Her-2, human epidermal growth factor receptor-2; CNB, core needle biopsy; SE, surgical excision

Considering the high prevalence of breast cancer in Iran, and the importance of hormonal receptors profile in such patients’ treatment outlook, the current study aimed at clarifying the hormonal profile of breast cancer specimens of both CNB and SE tissues. To do so, the study assessed 100 pairs of breast cancer cases of CNB pursued by surgical excisions.

The current study revealed that CNB and SE specimens had almost 90%, 81%, and 97.3% agreement for ER, PR, and Her-2immunohistochemical assessment. These findings were similar to those of the study by Arnedos et al., which demonstrated that CNB could be used with confidence for ER and Her-2 detection. For PR, they suggested that CNB should be used with caution. They studied 336 pairs of breast cancer cases and used SP1 and IE2 antibodies, and CB11 (Ventana, USA) for ER, PR, and Her-2, respectively. Assessment was done by Allred scoring. They presumed CNB as a reliable method to perform IHC staining (2).

Chem et al. (17),Omranipour et al. (4), and Zidan et al, also found similar results. In the latter research, the concordance rate for ER was estimated as 93%.The resemblance may be related to the use of monoclonal Ab 1D5(Dako)for ER (18).

TamKi et al. performed a comprehensive study on 353 breast cancer patients, using clone 6F11 (Ventana, Tucson, AZ, USA), clone 6 Ab (Ventana), and a standard immunohist-ochemistry(IHC) kit (Hercep Test for Immunoenzymatic Staining; Dako, Denmark)for ER,PR, and Her-2. They reported that concordance rate for ER and PR was 94.1% to 96% and 86.1% to 89.5%, respectively, while Her-2 had 64% to 96% concordance (1).Likewise, Tsuda et al. demonstrated that disagreement in the results may be related to intra-tumoralheterogeneity, pre-analytical factors including variation in the duration of tissue fixation and suboptimal processing, and borderline tumor properties. To overcome the problem of intra-tumoralheterogeneity, examination of large volume of tumor tissue appears to be necessary, and to solve the problem of borderline tumor properties in terms of Her-2 expression, judgment by multiple observers and DNA copy analysis might be of value (13). Some researchers also suggest that FISH test can be a help (12,13).

Lower correlation rates for PR compared to ER, could be explained by the focal and heterogeneous expression pattern of PR that can affect the results (19,20). In addition, PR is a highly sensitive receptor that can be damaged by inappropriate tissue fixation or processing methods (19). Besides, types of Ab, detection reagents, methods of interpretation, lack of technical standardization, and inter-laboratory reproducibility can influence the IHC assays (20). Therefore, pre-analysis conditions should be considered.

The current study had no case with CNB negative hormonal profile and SE positive results or vice versa, but there was one case that was weakly positive on CNB and strongly positive on SE; therefore the idea of decision-making separately about each patient was supported, and although the results showed the ability of CNB to predict patients' hormonal profile, at least the negative cases require more conservative interpretations. For surgical excisions, a delay between surgery and specimen delivery to a pathologic lab and a gap to pass the specimen can cause autolysis and advert the hormonal profile results (6,17,21).

Brenner et al. showed that for CNB, five specimens are enough to diagnose any lesion (22), but in the currentstudy found that the number of specimens had no significant effect on the concordance between CNB and SB to assess the hormonal status. 

## Conclusion

CNB can be performed confidently to determine ER, or Her-2 status of the tumors. For PR, results obtained from CNB should be utilized with consideration**.**
